# Animal Therapy in Tuberculosis

**Published:** 1904-05

**Authors:** Edward T. Smith

**Affiliations:** Buffalo, N. Y.


### Animal Therapy in Tuberculosis.

By EDWARD T. SMITH, M. D., Buffalo, N. Y.

ANIMAL therapeusis is now a well-defined therapy in many diseases, but up to the present time very little attention has been paid to the treatment of tuberculosis by the new remedies that fall within this category. By animal therapy I do not mean the use of the many serums and toxins that have been and are still used hypodermatically in this disease, or the internal administration of dessicated organic and glandular extracts. We have seen the rise and fall of so many serum and toxin cures for tuberculosis, that I wish to say now that I am not advocating any improvement or modification of those methods. The great danger in the use of toxins and the many disturbances of the digestive tract from the internal use of dessicated and liquid extracts have worked their own ruin in tuberculosis. Nothing can be said in favor of the chemical treatment by hypodermatic injection as the utter failure of Edson's aseptolin, gold, gold and manganese and many others testify.

It is, perhaps, treading upon dangerous ground to advise an entirely new way of treating this disease, but I have endeavored to fortify myself before so doing. I, in common with the great majority of physicians, have lost confidence in the many drugs and methods of treating tuberculosis and in the past years have turned to a new field in looking for a remedy,—the field of animal therapy. This field has been a fertile one for the many quack nostrums since the commencement of time, and has only been rehabilitated since the properties of the adrenal and thyroid glands have been discovered and used in a scientific manner.

The modern treatment of tuberculosis by supportive and hygienic measures is the most rational one and I only attempt to strengthen the method. From the Egyptian days of "henosis" to the present, all the investigators in the field of animal therapy have confined their efforts to obtain a remedy from testicular juice, either to be given hypodermatically or by the mouth. The Egyptian priests were far ahead of the present-day investigators, they injecting the finished product or semen of bulls per rectum. Testicular juice, per orem or hypodermatically, has little virtue as a remedy, while the injection of the finished product or semen in the compound, as I will later describe, has great medicinal properties as one constituent.

As the unit, I take the two-year-old bull, the nearest approach in the animal world to man in full vigor of health, an animal whose semen also is the nearest approach to human semen, and whose lymphatic and nervous systems are perfectly developed at this age, and by the process to be described, extract a lymph or cell tonic, a concentrated, vitalised, albuminous extract, representing the vitalising properties of the organs and secretions used in its preparation.

The mode of procedure is as follows: after tests to determine the healthy condition of the animal, he is closely confined for one month prior to making the cell tonic or lymph and fed freely upon grains to develop the lymphatics. During the month all the seminal fluid needful is obtained, which is preserved to add later to the compound prepared from the other organs and secretions. After thoroughly bleeding the animal to death, the gray cerebral substance, spinal cord, gangliæ, lymph in the reservoirs and ducts, mesenteric lymphatic glands are taken, and with the addition of the semen, the mixture is thoroughly triturated in a ball mill with an equal amount by weight of distilled water and half a grain of sublimed albuminate of gold to the ounce, added as a noncoagulant and preservative. The trituration must be carried on for ten hours continuously, which is easily done by connecting the mill to a motor. If after that time has elapsed, microscopical examination shows the entire disintegration of the spermatozoæ, lymphocytes, nerve cells and lymphatic pulp, the emulsion is filtered through a porous filter at a high pressure on account of its albuminous nature and afterward treated with ether or cold to remove the fats. It is then sterilised by carbonic acid gas under pressure. The lymph, as I call it, is a clear, thick albuminous liquid entirely free from fats and is a true extract of the organs and secretions before stated. No alcohol or glycerine can be used in its preparation.

I formerly used the lymph without filtration, injecting the emulsion, but found that the connective tissue, and the like, was inert and only a new danger for contamination. The product has no action in common with the so-called lymphs and extracts, animal or vegetable nucleins, dessicated extracts, or with the many forms of tonics placed upon the market for internal administration claiming to contain its essential constituents. Prepared with rigid antisepsis it is hard to contaminate, as are all mixtures containing seminal fluid, and will keep for months if placed in an amber colored glass stoppered bottle and kept in a dark, cool place. Any contamination is shown by an opalescence.

This compound is injected hypodermatically in tuberculosis to the exclusion of all other remedies, either local or general. The points of injection are the center and outer parts of each buttock, subscapular spaces, and along the sides of the spinal column. The injections are given deeply into the connective tissue, and not into the muscle, and leave no induration. I use a half-inch steel needle and the sub-Q glass syringe, and in giving the injections, I insert the needle its full length, slowly empty the syringe and, on withdrawal of needle, hold alcohol soaked gauze on the skin and make slight massage to spread the lymph and hasten absorption. The dangers to be avoided are superficial injections, haste, deficient massage, uncleanliness, and force in giving the injection. Force must not be used as it denotes injection into the muscle, a bloodvessel, or under a fascia. The lymph must flow freely with very slight pressure of the finger and if it does not do so, the needle should be withdrawn and reinserted. I do not use a given area too frequently as there is plenty of injecting surface, although I prefer the lower dorsal and lumbar regions.

Upon standing, the compound separates into two strata, hence, before drawing into the syringe, the liquid should be gently agitated, but it should not be made too frothy. If there is any pain from the injection it should be diluted with boiled or distilled water, but the injection is generally painless and in giving thousands of injections I never have had one ill result. The syringe is washed out with alcohol after each injection and with sterile or distilled water before injection. Nothing should be allowed to come in contact with the lymph except water, else it may coagulate.

The dosage varies in different individuals, according to the severity of the disease, location, and response to cell stimulus. In some severe cases of pulmonary tuberculosis it is necessary to give 10 minims every four to six hours until the severity of the symptoms abate, when the periods may be lengthened. In other cases not so acute I have had good results from one daily injection of 20 minims. If only one dose is given daily I give it in the early evening. The usual dose is 15 minims night and morning. There is such a thing as too energetic cell stimulation and the smaller the dose that gives results the better. This compound or any cell tonic that lessens or removes morbid processes by acting upon cell structure, function, and repair must have as an aid proper cell pabulum or food, and in using this therapy a most liberal diet of easily digested food is allowed, while beef juice, or whites of eggs taken in water or some light wine are used if forced feeding is necessary. Even if a patient's digestion is normal I insist on a plain, nutritious diet, excluding raw fruits as well as vegetables that leave much residue, such as cabbage, greens, beans, turnips, and the like.

The ideal food for these patients is raw scraped beef and whites of eggs, equal parts, made into meat balls and slowly fried in butter. This food is very easily digested and most patients seem to like it. I insist upon meats in abundance if patients can digest them, preferably beef, although I allow the easily digested vegetables. but no prepared foods. The natural meat fats and cream are called for if they do not produce stomach disturbances. If the patient has any aversion to hypodermatic injections I administer the lymph in hollow rectal suppositories. This can be done, also, if from any cause it is impossible to give the second daily injection, the suppository to be given at bedtime. As this treatment must be continued for some time I discontinue treatment every seventh day to prevent intolerance, if the patient is making progress. Clinical evidence shows that the great antagonists to the reconstructive action of this compound are: (1) products of indigestion; (2) constipation; (3) fatigue; (4) narcotics, alcohol in excess; (5) non-elimination; (6) venery.

Constipation is generally overcome by the tonic effect of the lymph upon the muscular coat of the bowels, but if not it is treated with the mildest remedies that have proved effective in my experience, but not with mercurials or salines. I insist upon rigid obedience in regard to the other conditions, especially as regards continence. Diluents, except in dilated stomach, salt glows to keep the skin active, pulmonary gymnastics, fresh air, and deep breathing exercises are active synergists to this therapy. Its use in tuberculosis in the manner in which I have described causes disappearance of bacilli, increases the number of leucocytes and red corpuscles, reduces fever and toxemia, eases cough and lessens expectoration, increases renal secretion, improves appetite and assimilation, causes absorption of consolidation and recent pleuritic adhesions, and cicatrises cavities. Of course the process is a gradual one on account of the reconstructive action of the compound and requires patience on the part both of physician and patient; but, as improvement generally commences very soon after institution of treatment, I have not had any trouble in that regard.

This lymph causes absorption of the induration surrounding cavities, thus allowing the blood charged with the natural antitoxin to come in direct contact with the diseased surfaces, destroying the bacilli and producing cicatrisation. The area of induration surrounding tubercular deposits and cavities is a feeble effort of the debilitated system to localise the disease, but it is practically degenerative, protecting the bacilli, and any remedy that improves the general health and removes this hyperplasia is beneficial. I will not further theorise upon its action and only claim the disease is cured in a natural manner by an extract which is a highly concentrated essence of the physical and nervous centers of an animal which is the nearest approach to man in the animal world. The lymph is a cell tonic and reconstructive simply.

In advanced cases in which there are large cavities and mixed infection with inhibited cell function from the long continued fever and toxemia, there may be small results from cell stimulation. The treatment should be used, in such cases, to prolong life and control certain symptoms, although I have seen some remarkable results where the cavities were not amphoric. In fibrous phthisis the treatment is used as a palliative, except in its incipiency, where often it is curative. The very simplicity of this therapy is much in its favor, and the absolute safety from the injection, the cleanliness and freedom from the continual drugging and dosing of nauseating medicines also commend it. Acting upon competent advice as to dosage and locality of injection, the lymph can be given by a nurse, one of the family, if properly instructed, or even in exceptional cases autoadministration by the patient may be permitted. The necessity of sending the patient away from the comfortable home and surroundings he or she is accustomed to, is obviated, the stomach and intestines are saved for the natural processes of digestion and assimilation, and the excreting surfaces of the kidneys are not impaired by the many phenols and other irritants given internally for this disease. The therapy, moreover, is applicable to all diseases of tubercular nature and, to some patients with a tubercular family history, I have given the treatment as a preventive, on account of its power to increase cell resistance.

I administer the lymph for three months, or longer if it seems advisable to do so, and the patient is kept under observation for a few months after it is discontinued. A number of my professional friends have employed this method and their experience only confirms my own. One of their patients had returned from the south in such a condition that he was confined to his bed, his sputum contained bacilli, and he also had empyema. In three months after commencing the treatment the patient was working at his usual occupation, practically cured. It is not an experimental therapy, but one that has proven its worth after years of clinical use and it appears to me to be an amplification of the most rational and scientific treatment of tuberculosis—namely, to increase cell resistance, improve hygiene, and promote reconstruction and repair.

The most discouraging case I ever treated under this plan was a man 32 years of age with well-defined pulmonary tuberculosis, having a cavity in the right apex and all the typical symptoms of this disease, complicated with secondary syphilis, presenting large patches of eruption upon the body, and besides, he had well-marked locomotor ataxia. In three months after the commencement of treatment all the symptoms of tuberculosis had disappeared, as also had the syphilitic manifestations and locomotor ataxia. The treatment consisted of one daily injection of 20 minims. This man has remained well for three years and has been working at a particularly hard kind of labor for that period. Any therapy that will arrest such a triad of diseases in one individual at the same time deserves consideration. It is, in my view, the superior treatment for incipient tuberculosis, doing away with the tedium and anxiety of many methods now in favor.

cutting short the further progress of the disease, and restoring the tedium of prolonged semiïnvalidism. My experience with this therapy in other infections and degenerative diseases will be presented at another time. A few cases of tuberculosis treated with this compound are summarised as follows:

H. M. Age 30. Consolidation of right apex, progressive emaciation and debility. Afternoon temperature 102°, expectoration scanty and contained tubercle bacilli; harassing cough and unable to work. First called to see him on account of severe pains in the head which attending physician thought meant tubercular meningitis. He was placed upon 20 minims of lymph twice daily. Pains in the head disappeared in less than two days, and after two months' treatment consolidated area in lung cleared up, bacilli disappeared, and the patient has remained well and at work ever since. This was five years ago.

J. F. Age 19. I was called to see this young man about three years ago to give my opinion as to the advisability of sending him to Phœnix, Ariz. I found him confined to his bed as the result of pulmonary hemorrhages and he was extremely anemic. A large cavity existed in the left lung, expectoration profuse, containing bacilli, temperature 103°, night sweats, pulse 130, no appetite and with all the symptoms of the later stage of tuberculosis. Under the circumstances it was suicidal to send him away, and I advised the use of the lymph compound. Placed him upon 20 minims twice daily and continued the treatment for three months. There was a gradual disappearance of all symptoms and now after three years there is no return of the disease.

D. M. Age 30. Consulted me in regard to cough, night sweats and increasing physical debility. Upon examination I found a cavity in the left apex, temperature 102°, expectoration profuse, containing bacilli and broken down lung tissue. Placed him upon one daily injection of 20 minims and one suppository at night containing the same amount; discharged him from treatment in ten weeks. Pulmonary cavity cicatrised, slight contraction of chest wall in left subscapular space, but no signs of tuberculosis. This was five years ago and patient has remained well, attending to his ordinary duties.

Mrs. C. Age 33. Persistent cough, diffuse bronchitis, great dulness over right apex and slight dulness over left, pulse 120, temperature 102°, no cavities but tubercle bacilli abundant in expectoration. Placed her upon one daily injection of 20 minims of lymph and improvement was immediate. Tubercle bacilli gradually disappeared from sputum and with the exception of slight dulness over right apex all the conditions are normal.

G. N. Age 19. Upon examination I found cavity in right apex with tubercular deposits; profuse expectoration, night sweats and rise of temperature to 103° ; expectoration loaded with tubercle bacilli. She came of a tubercular family, her mother, grandfather and four aunts dying of the disease. Placed her upon the compound and in one month the expectoration had greatly decreased, very few bacilli remained, appetite returned, no chills or sweats, cavity lessening and patient feeling comparatively well. She felt so well that nothing I said would influence her to continue treatment any longer than two more weeks. She should have continued the treatment for at least four months, as her history was very bad. However, she married and four years later died of acute tuberculosis.

Mrs. T. Age 36. Patient greatly emaciated, right apex consolidated, pulse 126, night sweats, temperature 101°, general bronchitis, and unable to go out of the house. Gave her two daily injections of 15 minims of lymph compound for one month, when she was compelled to leave the city. As it was impossible to continue her injections, I directed 20 minims twice daily by suppositories for two months longer, when she claimed there was no necessity for further treatment, as she was in her usual good health. This was two years ago and there has been no return of tuberculosis.

189 FOURTEENTH STREET.

